# Upconversion in solar cells

**DOI:** 10.1186/1556-276X-8-81

**Published:** 2013-02-15

**Authors:** Wilfried GJHM van Sark, Jessica de Wild, Jatin K Rath, Andries Meijerink, Ruud EI Schropp

**Affiliations:** 1 Copernicus Institute, Utrecht University, Budapestlaan 6, Utrecht 3584 CD, The Netherlands; 2 Physics of Devices, Debye Institute for Nanomaterials Science, Utrecht University, High Tech Campus 5, Eindhoven 5656 AE, The Netherlands; 3 Condensed Matter and Interfaces, Debye Institute for Nanomaterials Science, Utrecht University, P.O. Box 80000, Utrecht 3508 TA, The Netherlands; 4 Present address: Solar Energy, Energy research Centre of the Netherlands (ECN), High Tech Campus Building 5, p-057 (WAY), Eindhoven 5656 AE, The Netherlands; 5 Present address: Plasma & Materials Processing, Department of Applied Physics, Eindhoven University of Technology (TU/e), P.O. Box 513, Eindhoven 5600 MB, The Netherlands

**Keywords:** Upconversion, Photovoltaics, Thin-film silicon, Spectral modification, Lanthanides

## Abstract

The possibility to tune chemical and physical properties in nanosized materials has a strong impact on a variety of technologies, including photovoltaics. One of the prominent research areas of nanomaterials for photovoltaics involves spectral conversion. Modification of the spectrum requires down- and/or upconversion or downshifting of the spectrum, meaning that the energy of photons is modified to either lower (down) or higher (up) energy. Nanostructures such as quantum dots, luminescent dye molecules, and lanthanide-doped glasses are capable of absorbing photons at a certain wavelength and emitting photons at a different (shorter or longer) wavelength. We will discuss upconversion by lanthanide compounds in various host materials and will further demonstrate upconversion to work for thin-film silicon solar cells.

## Review

### Introduction

Attaining high conversion efficiencies at low cost has been the key driver in photovoltaics (PV) research and development already for many decades, and this has resulted in a PV module cost of around US$0.5 per watt peak capacity today. Some commercially available modules have surpassed the 20% efficiency limit, and laboratory silicon solar cells are getting closer and closer [1] to the Shockley-Queisser limit of 31% for single-junction silicon cells [2]. However, a fundamental issue is that conventional single-junction semiconductor solar cells only effectively convert photons of energy close to the bandgap (*E*_g_) as a result of the mismatch between the incident solar spectrum and the spectral absorption properties of the material [3]. Photons with energy (*E*_ph_) smaller than the bandgap are not absorbed, and their energy is not used for carrier generation. Photons with energy (*E*_ph_) larger than the bandgap are absorbed, but the excess energy *E*_ph_ – *E*_g_ is lost due to thermalization of the generated electrons. These fundamental spectral losses are approximately 50% [4]. Several approaches have been suggested to overcome these losses, e.g., multiple stacked cells [5], intermediate bandgaps [6], multiple exciton generation [7], quantum dot concentrators [8,9], and spectral converters, the latter being down- and upconverters [10,11] and downshifters [12,13]. In these so-called *third- or next-generation PV* concepts [14,15], nanotechnology is deemed essential in realizing most of these concepts [16].

#### Spectral conversion

Spectral conversion aims at modifying the incident solar spectrum such that a better match is obtained with the wavelength-dependent conversion efficiency of the solar cell. Its advantage is that it can be applied to existing solar cells and that optimization of the solar cell and spectral converter can be done separately. Different types of spectral conversion can be distinguished: (a) upconversion, in which two low-energy (sub-bandgap) photons are combined to give one high-energy photon; (b) downshifting or luminescence, in which one high-energy photon is transformed into one lower energy photon; and (c) downconversion or quantum cutting, in which one high-energy photon is transformed into two lower energy photons. Downshifting can give an efficiency increase by shifting photons to a spectral region where the solar cell has a higher quantum efficiency, i.e., basically improving the blue response of the solar cell, and improvements of up to 10% relative efficiency increase have been predicted [13]. Up- and downconversion, however, are predicted to be able to raise the efficiency above the SQ limit [10,11]. For example, Richards [12] has shown for crystalline silicon (c-Si) that the potential relative gain in efficiency could be 32% and 35% for downconversion and upconversion, respectively, both calculated for the standard 1,000-W/m^2^ air mass (AM) 1.5 solar spectrum.

Research on spectral conversion is focused on organic dyes, quantum dots, lanthanide ions, and transition metal ion systems for up- and downconversion [13,17,18]. An upconversion layer is to be placed at the back of the solar cells, and by converting part of the transmitted photons to wavelengths that can be absorbed, it is relatively easy to identify a positive contribution from the upconversion layer, even if the upconversion efficiency is low. In contrast, proof-of-principle experiments in solar cells are complicated for downconverters and downshifters because of the likelihood of competing non-radiative processes. These downconverters and downshifters have to be placed at the front of the solar cell, and any efficiency loss will reduce the overall efficiency of the system.

**Figure 1:**
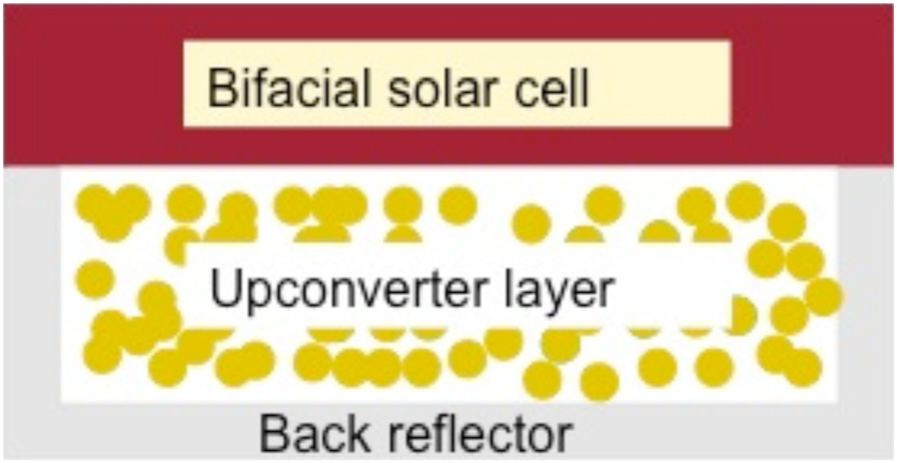
**Schematic view of solar cell with upconverter layer at the back.** It is surrounded by a back reflector to ensure that upconverted radiation is directed towards the solar cell where it can be absorbed.

Downconversion with close to 200% internal quantum efficiency has been demonstrated, but the actual quantum efficiency is lower due to concentration quenching and parasitic absorption processes [19,20]. Even for a perfect 200% quantum yield system, a higher solar cell response requires a reflective coating to reflect the isotropically emitted photons from the downconversion layer back towards the solar cell. However, no proof-of-principle experiments have been reported to demonstrate an efficiency gain using downconversion materials. An upconverter also emits isotropically, but since it is placed at the back of the solar cells, the upconversion photons can easily be directed into the solar cell by placing a reflector behind the upconverter layer, as depicted in Figure 1.

The usefulness of down- and upconversion and downshifting depends on the incident spectrum and intensity. While solar cells are designed and tested according to the ASTM standard [21], these conditions are rarely met outdoors. Spectral conditions for solar cells vary from AM0 (extraterrestrial) via AM1 (equator, summer and winter solstice) to AM10 (sunrise, sunset). The weighted average photon energy (APE) [22] can be used to parameterize this; the APE (using the range 300 to 1,400 nm) of AM1.5G is 1.674 eV, while the APE of AM0 and AM10 are 1.697 and 1.307 eV, respectively. Further, overcast skies cause higher scattering leading to diffuse spectra, which are blue-rich, e.g., the APE of the AM1.5 diffuse spectrum is calculated to be 2.005 eV, indeed much larger than the APE of the AM1.5 direct spectrum of 1.610 eV. As downconversion and downshifting effectively red-shift spectra, the more relative energy an incident spectrum contains in the blue part of the spectrum (high APE), the more gain can be expected [12,23]. Application of downconversion layers will therefore be more beneficial for regions with high diffuse irradiation fraction, such as Northwestern Europe, where this fraction can be 50% or higher. In contrast, solar cells with upconversion (UC) layers will be performing well in countries with high direct irradiation fractions or in early morning and evening due to the high air mass resulting in low APE, albeit that the non-linear response to intensity may be limiting. Up- and downconversion layers could be combined on the same solar cell to overcome regionally dependent efficiencies. Optimization of either up- or downconversion layers could be very effective if the solar cell bandgap is a free design parameter.

In this paper, we focus on upconversion materials for solar cells, in particular for thin-film silicon solar cells. We describe the present state of the art in upconversion materials and application in solar cells.

### Upconversion

#### Principles

Upconversion was suggested by Bloembergen [24] and was related to the development of infrared (IR) detectors: IR photons would be detected through sequential absorption, as would be possible by the arrangement of energy levels of a solid. However, as Auzel pointed out, the essential role of energy transfer was only recognized nearly 20 years later [25]. Several types of upconversion mechanism exist, of which the *addition de photon par transferts d’energie* or, in English, energy transfer upconversion mechanism is the most efficient; it involves energy transfer from an excited ion, named sensitizer, to a neighboring ion, named activator [25]. Others are two-step absorption, being a ground-state absorption followed by an excited-state absorption, and second-harmonic generation. The latter mechanism requires extremely high intensities, of about 10^10^ times the sun’s intensity on a sunny day, to take place [26] and can therefore be ruled out as a viable mechanism for solar cell enhancement.

Upconverters usually combine an active ion, of which the energy level scheme is employed for absorption, and a host material, in which the active ion is embedded. The most efficient upconversion has been reported for the lanthanide ion couples (Yb, Er) and (Yb, Tm) [27]. The first demonstration of such an upconversion layer was reported by Gibart et al. [28] who used a GaAs cell on top of a vitroceramic containing Yb^3+^ and Er^3+^: it showed 2.5% efficiency under very high excitation densities.

#### Upconverter materials

**Figure 2:**
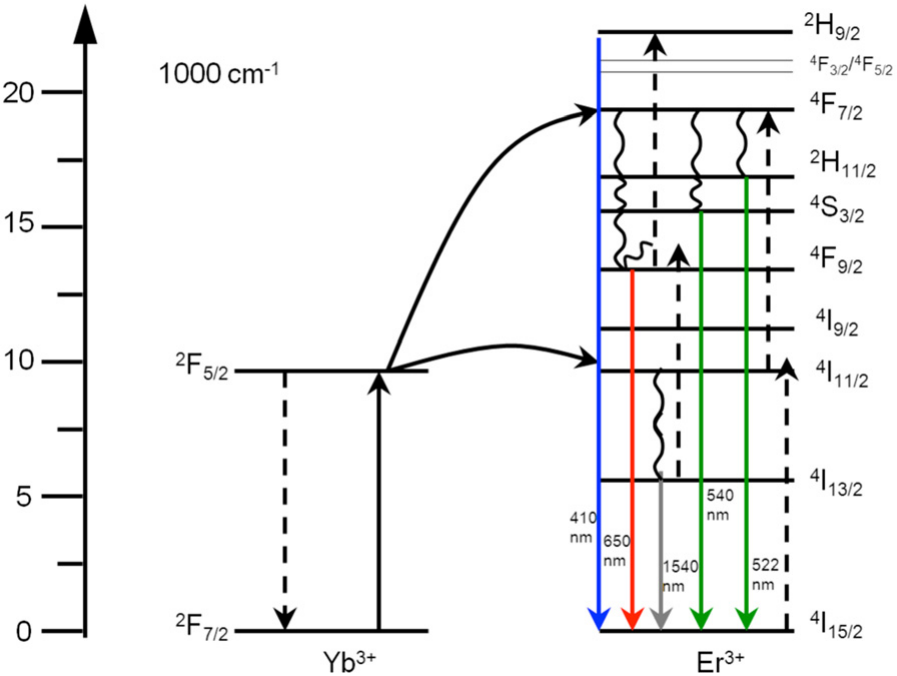
**Upconversion in the (Yb**^**3+**^**, Er**^**3+**^**) couple.** The dashed lines represent energy transfer, the full lines represent the radiative decay, and the curly lines indicate multi-phonon relaxation processes. The main route is a two-step energy transfer after excitation around 980 nm in the Yb^3+^ ion that leads to excitation to the ^4^F_7/2_ state of the Er^3+^ ion. After relaxation from this state, emission is observed from the ^2^H_11/2_ level, the ^4^S_3/2_ level (green), and the ^4^F_9/2_ level (red).

Lanthanides have been employed in upconverters attached to the back of bifacial silicon solar cells. Trivalent erbium is ideally suited for upconversion of near-infrared (NIR) light due to its ladder of nearly equally spaced energy levels that are multiples of the ^4^I_15/2_ to ^4^I_13/2_ transition (1,540 nm; see also Figure 2). Shalav et al. [29] have demonstrated a 2.5% increase of external quantum efficiency due to upconversion using NaYF_4_:20% Er^3+^. By depicting luminescent emission intensity as a function of incident monochromatic (1,523 nm) excitation power in a double-log plot, they showed that at low light intensities, a two-step upconversion process (^4^I_15/2_ → ^4^I_13/2_ → ^4^I_11/2_) dominates, while at higher intensities, a three-step upconversion process (^4^I_15/2_ → ^4^I_13/2_ → ^4^I_11/2_ → ^4^S_3/2_level) is involved.

Strümpel et al. have identified the materials of possible use in up- (and down-) conversion for solar cells [26]. In addition to the NaYF_4_:(Er,Yb) phosphor, they suggest the use of BaCl_2_:(Er^3+^,Dy^3+^) [30], as chlorides were thought to be a better compromise between having a low phonon energy and a high-excitation spectrum, compared to the NaYF_4_[31,32]. These lower phonon energies lead to lower non-radiative losses. In addition, the emission spectrum of dysprosium is similar to that of erbium, but the content of Dy^3+^ should be <0.1% to avoid quenching [25,26].

NaYF_4_ co-doped with (Er^3+^, Yb^3+^) is, to date, the most efficient upconverter [27,33], with approximately 50% of all absorbed NIR photons upconverted and emitted in the visible wavelength range. However, the (Yb, Er) couple is not considered beneficial for upconversion in c-Si cells as silicon also absorbs in the 920- to 980-nm wavelength range. These phosphors can be useful for solar cells based on higher bandgap materials such as the dye-sensitized solar cell (DSSC) or Grätzel cell [34], a-Si(Ge):H, or CdTe. Different mechanisms are responsible for the upconversion luminescence. The Yb^3+^ ion has only one excited state and is an ideal sensitizer for Er^3+^ because of the relatively high oscillator strength of the ^2^F_7/2_ → ^2^F_5/2_ transition and the fact that Er^3+^ has a state with similar energy (^4^I_11/2_) which is populated by energy transfer from Yb^3+^ (see Figure 2). Population of the first excited state of Er^3+^ (^4^I_11/2_) is therefore directly proportional to the incoming light intensity. When upconversion is the main route, energy transfer from the first excited state (^4^I_11/2_) to the second excited state (^4^F_7/2_) follows. After some small energy-relaxation steps, emission is observed from the ^4^S_3/2_, ^2^H_11/2_ (green), and ^4^F_9/2_ (red) states. The ^4^F_9/2_ can also be reached after energy transfer from the ^4^I_13/2_ state.

As two or more photons are required for upconverted emission, a higher order dependence of the incoming light intensity is expected:

(1) $$N_{n}\propto N_{n-1}N_{s}\propto {\left(N_{s}\right)}^{n}\propto {P_{\mathrm{in}}}^{n}\text{,}$$

where *n* is the number of photons needed to excite the upconverted state. *N*_*n*_ is the *n*th excited state in the Er^3+^ ion, and *N*_s_ is the excited state of the sensitizer ion Yb^3+^. When a higher energy level saturates, other processes like non-radiative relaxation to lower energy states occur, and as a consequence, deviations from the expected power law dependence are observed [35,36]. The upconverted emission intensity is thus proportional to the population of the higher excited state *N*_*n*_. When an upconverter is applied to the back of a solar cell, the increased photogenerated current is due to this emission, and thus,

(2) $$I_{\mathrm{SC}}^{\mathrm{UC}}\propto P_{\mathrm{in}}^{n}$$

where *P*_in_ is the incoming light intensity and *I*_SC_^UC^ is the photogenerated short-circuit current increase due to upconversion in the solar cell. As a result, for current increase due to upconversion, a quadratic power dependence on the concentration factor is expected.

De Wild et al. recently applied a commercially available upconverter, Gd_2_O_2_S:Yb^3+^, Er^3+^, in which Yb^3+^ absorbs light around 980 nm and Er^3+^ emits in the visible spectrum (400 to 700 nm) [37]. These absorption and emission wavelengths are very suitable for use with wide-bandgap solar cells, such as single-junction a-Si:H, as the absorption edge of a-Si:H is between the wavelengths for absorption and emission. Furthermore, the spectral response is very high in that emission range. The dominant upconversion mechanism in Gd_2_O_2_S:Yb^3+^, Er^3+^ is energy transfer upconversion.

Nanocrystals of NaYF_4_:Er^3+^, Yb^3+^ also show upconversion. An advantage of using nanocrystals is that transparent solutions or transparent matrices with upconverting nanocrystals can be obtained. Recent reviews on upconverting nanoparticles summarize the status of a variety of upconverter materials that are presently available as nanocrystals, mostly phosphate and fluoride nanocrystals [38,39]. However, a problem with upconversion nanocrystals is the lower upconversion efficiency [40]. There is a clear decrease in efficiency with decreasing size in the relevant size regime between 8 and 100 nm, which is probably related to surface effects and quenching by coupling with high-energy vibrations in molecules attached to the surface.

Upconversion systems consisting of lanthanide nanocrystals of YbPO_4_ and LuPO_4_ have been demonstrated to be visible by the naked eye in transparent solutions, however at efficiency lower than that of solid-state upconversion phosphors [27]. Other host lattices (Na_X_F_4_, X = Y, Gd, La) have been used, and co-doping with Yb^3+^ and Er^3+^, or Yb^3+^ and Tm^3+^ appeared successful, where Yb^3+^ acts as sensitizer. Nanocrystals of <30 nm in size, to prevent scattering in solution, have been prepared, and they can be easily dissolved in organic solvents forming colloidal solutions, without agglomeration. Further efficiency increase is possible by growing a shell of undoped NaYF_4_ around the nanocrystal; in addition, surface modification is needed to allow dissolution in water, for use in biological labeling.

Porous silicon layers are investigated for use as upconverter layers as host for rare-earth ions because these ions can easily penetrate the host due to the large surface area and porosity. A simple and low-cost dipping method has been reported [41], in which a porous silicon layer is dipped into a nitrate solution of erbium and ytterbium in ethanol (Er(NO_3_)_3_:Yb(NO_3_)_3_:C_2_H_5_OH), which is followed by a spin-on procedure and a thermal activation process at 900°C. Excitation of the sample at 980 nm revealed upconversion processes as visible and NIR photoluminescence is observed; co-doping of Yb with Er is essential, and doping only with Er shows substantial quenching effects [42].

Finally, sensitized triplet-triplet annihilation (TTA) using highly photostable metal-organic chromophores in conjunction with energetically appropriate aromatic hydrocarbons has been shown to be another alternative upconversion system [43,44]. This mechanism was shown to take place under ambient laboratory conditions, i.e., low-light-intensity conditions, clearly of importance for outdoor operation of solar cells. These chromophores (porphyrins in this case) can be easily incorporated in a solid polymer such that the materials can be treated as thin-film materials [45]. A problem with TTA upconverters is the spectral range. No efficient upconversion of NIR radiation at wavelengths beyond 800 nm has been reported which limits the use to wide-bandgap solar cells [37,46].

### Upconversion for solar cells

#### Efficiency limits

Upconversion in solar cells was calculated to potentially lead to a maximum conversion efficiency of 47.6% [11] for nonconcentrated sunlight using a 6,000-K blackbody spectrum in detailed-balance calculations. This optimum is reached for a solar cell material of approximately 2-eV bandgap. Applied on the back of silicon solar cells, the efficiency limit would be approximately 37% [11]. The analysis of the energy content of the incident AM1.5G spectrum shows that cells with an upconverter layer would benefit from an extra amount of 35% light incident in the silicon solar cell [12]. An extension to the models described above was presented in a study by Trupke et al. [47], in which realistic spectra were used to calculate limiting efficiency values for upconversion systems. Using an AM1.5G spectrum leads to a somewhat higher efficiency of 50.69% for a cell with a bandgap of 2.0 eV. For silicon, the limiting efficiency would be 40.2% or nearly 10% larger than the value of 37% obtained for the 6,000-K blackbody spectrum [11]. This increase was explained by the fact that absorption in the earth’s atmosphere at energies lower than 1.5 eV (as evident in the AM1.5G spectrum) leads to a decrease in light intensity. Badescu and Badescu [48] have presented an improved model that takes into account the refractive index of solar cell and converter materials in a proper manner. Two configurations are studied: cell and rear converter, the usual upconverter application, and front converter and cell (FC-C). They confirm the earlier results of Trupke et al. [11] in that the limiting efficiency is larger than that of a cell alone, with higher efficiencies at high concentration. Also, the FC-C combination, i.e., upconverter layer on top of the cell, does not improve the efficiency, which is obvious. Further, building on the work by Trupke et al. [11], the variation of refractive indexes of cell and converter was studied, and it was found that the limiting efficiency increases with the refractive index of both cell and upconverter. In practice, a converter layer may have a lower refractive index (1.5, for a transparent polymer: polymethylmethacrylate (PMMA) [49]) than a cell (3.4). Using a material with a similar refractive index as the cell would improve the efficiency by about 10%. Finally, a recent study on realistic upconverter and solar cell systems, in which non-ideal cell and upconverters were considered, corroborates the above findings [50]. In this study, non-ideal absorption and radiative recombination, as well as non-radiative relaxation in the upconverter, were taken into account. Atre and Dionne also stressed that thin-film PV with wide-bandgap materials may benefit the most from including upconverters [50].

#### Experiments

The first experiment in which an upconversion layer was applied on the back of solar cells comprised an ultrathin (3 μm) GaAs cell (bandgap 1.43 eV) on top of a 100-μm-thick vitroceramic containing Yb^3+^ and Er^3+^[28]: it showed 2.5% efficiency upon excitation of 256-kW/m^2^ monochromatic sub-bandgap (1.391 eV) laser light (1 W on 0.039-cm^2^ cell area) as well as a clear quadratic dependence on incident light intensity. An efficiency of the solar cell of 2.5% was obtained even though the excitation wavelength (891 nm) is not resonant with the absorption peak of Yb^3+^ (approximately 980 nm), leading to inefficient upconversion. Secondly, the design was such that not all emitted photons were directed to the solar cell.

Richards and Shalav [51] showed upconversion under a lower excitation density of 2.4 W*/*cm^2^ reaching 3.4% quantum efficiency at 1,523 nm in a crystalline silicon solar cell with NaYF_4_ doped with Er^3+^ as upconverter. This was for a system optimized for the wavelength of 1,523 nm. Intensity-dependent measurements showed that the upconversion efficiency was approaching its maximum due to saturation effects [51,52]. Under broadband excitation, upconversion was shown for the same system by Goldschmidt et al. [53] reaching an upconversion efficiency of 1%. Since c-Si has a rather small bandgap (1.12 eV), transmission losses due to the low energy photons are not as high as for wider bandgap solar cells. Hence, the efficiency gain for larger bandgap solar cells is expected to be higher. Upconversion of 980-nm light was also demonstrated in DSSCs [54,55] and of 750-nm light in ultrathin (50 nm) a-Si:H solar cells in 2012 [56]. In the latter proof-of-principle experiment, for the first time, an organic upconverter was applied.

### Upconversion for a-Si:H solar cells

**Figure 3:**
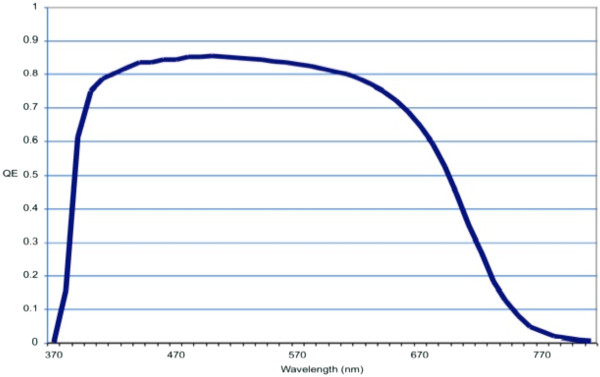
Typical spectral response of a-Si:H solar cells (courtesy of JW Schüttauf).

A typical external collection efficiency (ECE) graph of standard single-junction p-i-n a-Si:H solar cells is shown in Figure 3. These cells are manufactured on textured light-scattering SnO_2_:F-coated glass substrates and routinely have >10% initial efficiency. Typically, the active Si layer in the cells has a thickness of 250 nm, and the generated current is 14.0 to 14.5 mA/cm^2^, depending on the light-trapping properties of the textured metal oxide and the back reflector. After light-induced creation of the stabilized defect density (Staebler-Wronski effect [57]), the stabilized efficiency is approximately 9%. From Figure 3, it can be seen that the maximum ECE is 0.85 at approximately 550 nm, and the cutoff occurs at approximately 700 nm, with a response tailing towards 800 nm. The purpose of an upconverter is to tune the energy of the emitted photons to the energy where the spectral response shows a maximum. If the energy of the emitted photons is too close to the absorption limit (the bandgap edge), then the absorption is too low and the upconverted light would not be fully used.

The photogenerated current could be increased by 40% if the spectral response was sustained at high level up to the bandgap cutoff at 700 nm and by even more if light with wavelengths *λ* > 700 nm could be more fully absorbed. These two effects can be achieved with the upconversion layer, combined with a highly reflecting back contact. While the upconversion layer converts sub-bandgap photons to ‘super’-bandgap photons that can thus be absorbed, a non-conductive reflector is a much better alternative than any metallic mirror, thus sending back both the unabsorbed super-bandgap photons as well as the upconverted super-bandgap photons into the cell. It is commonly estimated that the stabilized efficiency of the approximately 9% cell can be enhanced to approximately 12%. Besides a-Si, a material denoted as protocrystalline Si could be used; this is an amorphous material that is characterized by an enhanced medium-range structural order and a higher stability against light-induced degradation compared to standard amorphous silicon. The performance stability of protocrystalline silicon is within 10% of the initial performance; its bandgap is slightly higher than that of amorphous silicon.

De Wild et al. [58] have demonstrated upconversion for a-Si cells with NaYF_4_ co-doped with (Er^3+^, Yb^3+^) as upconverter. The upconverter shows absorption at 980 nm (by the Yb^3+^ ion) leading to efficient emission of 653- (red) and 520- to 540-nm (green) light (by the Er^3+^) after a two-step energy transfer process. The narrow absorption band around 980 nm for Yb^3+^ limits the spectral range of the IR light that can be used for upconversion. An external quantum efficiency of 0.02% at 980-nm laser irradiation was obtained. By using a third ion (for example, Ti^3+^) as a sensitizer, the full spectral range between 700 and 980 nm can be efficiently absorbed and converted to red and green light by the Yb-Er couple. A transition metal ion such as Ti^3+^ incorporated in the host lattice absorbs over a broad spectral region and transfers the energy to a nearby Yb^3+^ ion through a dipole-dipole interaction [27,31]. The resulting light emission in the green and red regions is very well absorbed by the cell with very good quantum efficiency for electron–hole generation.

#### Bifacial solar cells with upconverter

Concentrated broadband light excitation has recently been used to study two types of bifacial a-Si:H solar cells that were made with and without Gd_2_O_2_S:Er^3+^, Yb^3+^ upconverter attached at the back of the cells [59]. The upconverter powder mixture was applied to the rear of the solar cells by first dissolving it in a solution of PMMA in chloroform, after which it was drop cast. Two types of p-i-n a-Si:H solar cells were made: one on Asahi-textured SnO2:F glass and one on flat ZnO:Al 0.5% superstrate. The efficiency obtained for the cells is 8% for textured and 5% for flat solar cells, both without a back reflector. Backside illumination yields an efficiency of 5% for textured solar cells and 4% for flat solar cells. With illumination from the back, the efficiency is lower because the generation profile is reversed within the cell, and thus, the photogenerated minority carriers have to travel the largest mean distance, rather than the majority carriers. The spectral response measured through the n-layer shows a quantum efficiency of 0.7 for both textured and flat solar cells at 550 nm; the spectral response at 660 nm is lower, i.e., 0.4 for textured cells and 0.15 for flat cells. The transmission for 900 to 1,040 nm was 40% to 45% for the textured solar cells and between 60% and 80% for the flat solar cells. The thickness of the i-layer was chosen such that an interference maximum occurs at 950 nm, increasing the transmission at this wavelength. As a result, more light can be absorbed by the upconverter layer in the case of the flat solar cell configuration. Concentration levels of up to 25 times were reached using near-infrared light from a solar simulator.

**Figure 4:**
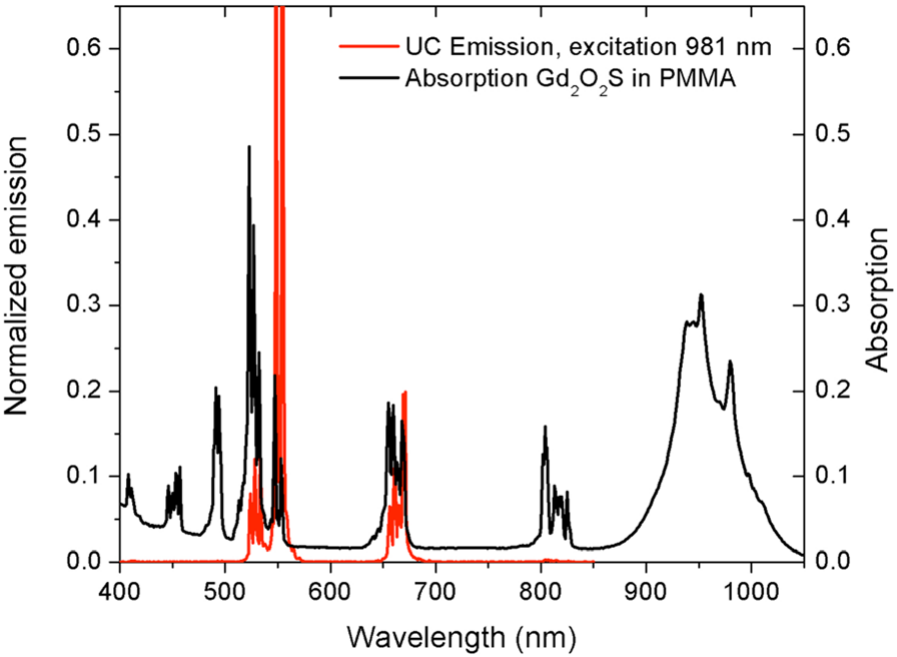
**Upconverted emission and absorption spectra of the upconverter in PMMA layer.** The emission spectrum is obtained when the upconverter shows no saturation and only emission peaks from the ^4^S_3/2_, ^2^H_11/2_ (510 to 560 nm), and ^4^F_9/2_ (650 to 680 nm) states are observed.

The absorption and emission spectra of the upconverter are shown in Figure 4. The absorption is highest around 950 nm. The upconverter was excited with filtered light of a xenon lamp at 950 ± 10 and 980 ± 10 nm. The ^4^F_7/2_ state at 2.52 eV is reached after two energy transfer events from Yb to Er. The upconverter was already shown to be very efficient at low light intensities. Saturation was measured under light intensities of less than 1 W/cm^2^. Although the absorption at 950 nm (1.31 eV) is higher, excitation at 980 nm (1.26 eV) leads to two times higher upconverted emission intensity. This may be attributed to the perfectly resonant energy transfer step of 980 nm (1.26 eV) since the ^4^F_7/2_ state is at 2.52 eV.

**Figure 5:**
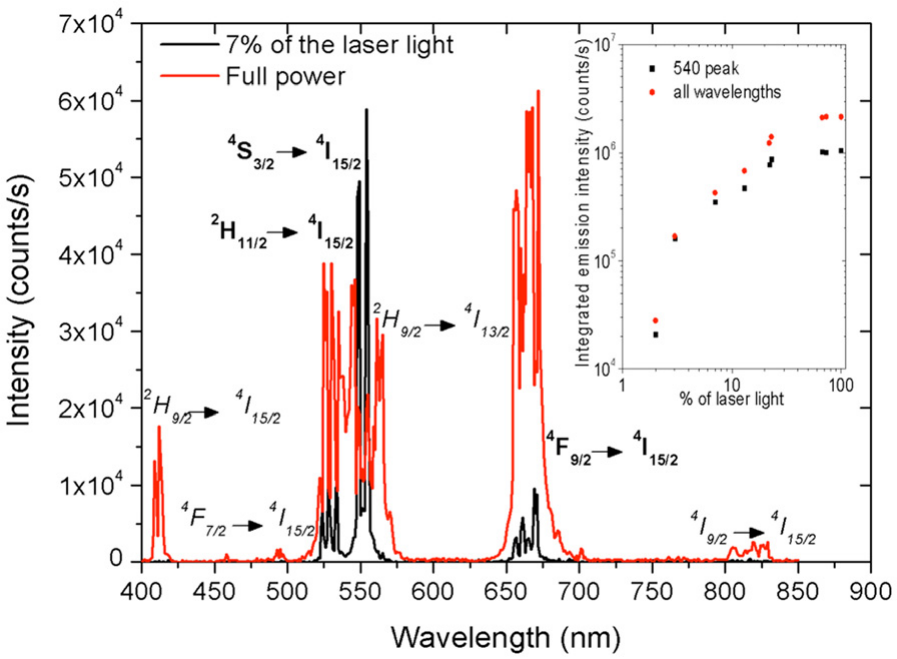
**Upconverted emission spectra under low and high excitation density.** For the low excitation power, the green state was not yet saturated. The intensities may be compared. New peaks (italic) are assigned: ^2^H_9/2_ → ^4^I_15/2_ transition at 410 nm, ^4^I_9/2_ → ^4^I_15/2_ transition at 815 nm, and the intermediate transition ^2^H_9/2_ → ^4^I_13/2_ at 560 nm.

For further experiments, the upconverter was excited at 980 nm with a pulsed Opotek Opolette laser. Because upconversion is a two-photon process, the efficiency should be quadratically dependent on the excitation power density. The intensity of the laser light was varied with neutral density filters. Upconversion spectra were recorded in the range of 400 to 850 nm under identical conditions with varying excitation power. Varying the intensity shows that for low light intensities, the red part is less than 6% of the total emission (see Figures 4 and 5). Only when the emission from the green-emitting states becomes saturated does the red emission become more significant and even blue emission from the ^2^H_9/2_ state is measured (see Figure 5). By comparing the emission intensities, it becomes clear that the emission intensity is not increasing quadratically with excitation power density. Instead, emissions from higher and lower energy states are visible. The inset in Figure 5 shows the integrated emission peaks for the green and total emissions, showing that at very high laser intensities, the total emission is saturated.

#### Sub-bandgap response

**Figure 6:**
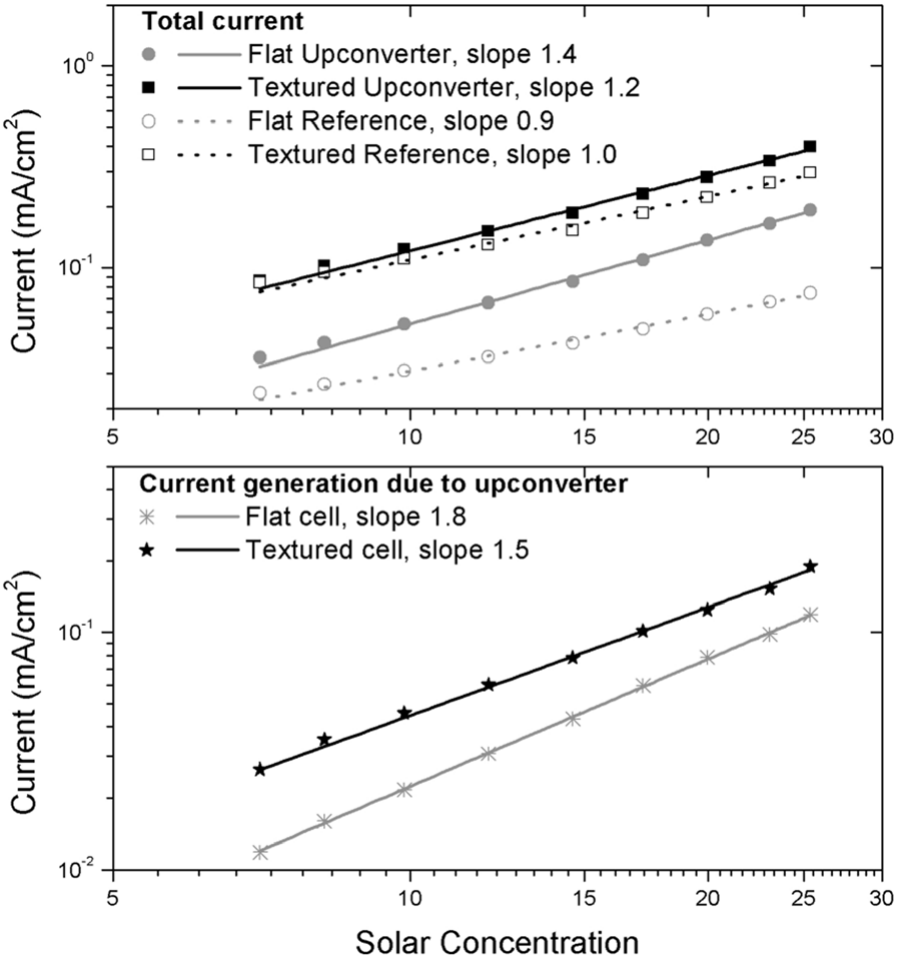
**Current measured in the solar cells under illumination of sub-bandgap light.** In the upper graph, the total current of the reference and UC cells are plotted as a function of the concentration factor, while in the lower graph, the current generated by the upconverter is shown. The slope for sub-bandgap response is 1 for flat and textured solar cells. The contribution of the upconverter increases the slope slightly; when corrected for the sub-bandgap response, the slope is 1.5 for the textured solar cells and 1.8 for the flat solar cells.

The sub-bandgap response in the near infrared due to the band tails of a-Si:H solar cells cannot be neglected [58]. To distinguish between upconverter response and sub-bandgap response, intensity-dependent current–voltage measurements are performed on solar cells with and without an upconverter at wavelengths longer than 900 nm using a solar simulator and a 900-nm-long pass filter. Intrinsic response of the band tails is linearly dependent on the light intensity, while response due to upconverted light is expected to be quadratically increasing with the concentration. Figure 6 shows the current measured for the different solar cells with different concentration factors of the sub-bandgap light. The slope of the line fitted to the data yields the value *n*, as given by Equation 2. As expected, the sub-bandgap response linearly increases with light intensity and values of *n* larger than 1 are measured for the upconversion solar cells. Note that the value is rather close to 1 because a large part of the total current is due to the sub-bandgap response (see Figure 6, upper graph). When the total current measured for the upconverter solar cells is corrected for the sub-bandgap response, the current due to upconversion only shows a higher value for *n* (see Figure 6, lower graph), i.e., a value of *n* = 1.5 and *n* = 1.8 is determined for textured and flat solar cells, respectively. Clearly, the current is not increasing quadratically with increasing concentration. It is unlikely that the upconverter is saturated because the power density is far below the saturation level of 0.6 W/cm^2^. It is therefore more likely that the deviations are due to decreasing carrier collection efficiency with increasing concentration. This effect would play a larger role in textured solar cells because they have a higher defect density than flat solar cells. This may explain why the value of *n* is closer to 2 for flat solar cells than for textured solar cells.

#### Narrow and broadband light comparison

Monochromatic laser light with wavelength at 981 nm and a power density of 0.2 W/cm^2^ was used for textured solar cells and yielded a current density of 0.14 mA/cm^2^ for the upconverter solar cells and 0.04 mA/cm^2^ for the reference solar cells. Evidently, the contribution of sub-bandgap absorption is much smaller using monochromatic laser light. The current due to the upconverter is comparable to the current measured under 20 sun: approximately 0.1 mA/cm^2^ (see Figure 6). This is remarkable in two ways. First, the results are in contrast with previously reported experiments with broadband excitation of c-Si solar cells [53], where the current under broadband excitation was much smaller than that under laser light excitation. However, in [53], another upconverter was applied (NaYF_4_) and different processes occur in the upconverter, namely excited state absorption. In the upconverter in this work (Gd_2_O_2_S), energy transfer upconversion is the main upconversion path, and the broadband absorption of Yb^3+^ may increase the transfer between Yb^3+^ and Er^3+^.

Second, the power that is absorbed by Yb^3+^ is 3.44 mW/cm^2^[37], which yields a broadband power density of 70 mW/cm^2^ under a concentration of 20 sun. This is three times less than the power density of the laser. A large difference here is that for broadband illumination, a 900-nm-long pass filter was used. Therefore, light of the solar simulator extends to further than 1,600 nm; thus, also the ^4^I_13/2_ state of Er^3+^ is excited directly. Addition of other paths that lead to upconverted light may contribute to the current. These paths may be non-resonant excited-state absorption between the energy levels of Er^3+^ or three-photon absorption around 1,540 nm at the ^4^I_13/2_ state of Er^3+^ (see Figure 2). Direct excitation of the ^4^I_13/2_ state of Er^3+^ followed by excited-state absorption from ^4^I_13/2_ to ^2^F_9/2_ results in a visible photon around 650 nm, while three-photon absorption around 1,540 nm results in emission from the ^2^F_9/2_ state too. Wavelengths required for these transitions are around 1,540 and 1,200 nm, which are present within the broad excitation spectrum. Contribution of these upconversion routes increases the emission and thereby the current in the solar cells.

### Outlook

Upconversion for solar cells is an emerging field, and the contribution of upconverter research to upconverter solar cell research increases rapidly. However, up to now, only proof-of-principle experiments have been performed on solar cells, mainly due to the high intensities that are deemed necessary. Some routes to enhance absorption are presently being developed, such as external sensitization and plasmonics.

External sensitization can be achieved by, e.g., quantum dots or plasmons. Quantum dots (QDs) can be incorporated in a concentrator plate where the QDs absorb over a broad spectral range in the IR and emit in a narrow line, e.g., around 1,520 nm, resonant with the Er^3+^ upconversion wavelength. Energy transfer from the QDs to Er^3+^ in this scheme is through radiative energy transfer. The viability of this concept was proven by Pan et al. [60] in c-Si solar cells, where a layer with QDs was placed below the upconverter layer. With the QDs, more light was absorbed and upconverted, which was proven by measuring the excitation spectra for the upconverted emission. The increased upconverted emission resulted in higher currents in the solar cell.

More challenging are options to enhance upconversion efficiencies by manipulating emission and excitation processes through plasmonic coupling [61]. The use of plasmonic effects with upconverter materials is a new and emerging field, with many possibilities and challenges. In general, plasmonic resonance can be used in two ways to increase the upconversion efficiency: by enhancing either the absorption strength or the emission strength. When the absorption strength is enhanced, the emission increases with the square of the enhancement in the non-linear regime. In the case of resonance between the plasmon and the optical transition, strong enhancement can be achieved. Recently, Atre et al. [62] have modelled the effects of a spherical nanocresent consisting of a core of an upconverter material and a crescent-shaped Ag shell. A 10-fold increase in absorption as well as a 100-fold increase in above-bandgap power emission toward the solar cell was calculated. A similar study has been performed using Au nanoparticles [63]. Experimental proof has recently been reported by Saboktakin et al. [64]. A related method is to enhance the absorption strength by nanofocusing of light in tapered metallic structures [65]. At the edges, enhancement has been reported due to focusing of the light in these areas. The other option is enhancing the emission. In this case, the emission of the upconverter is enhanced by nearby plasmon resonances [66]. Since the field enhancement decays away exponentially with the distance to metallic nanoparticle, the upconverter species have to be close to the surface of the nanoparticle to benefit from the field enhancement effects. For organic molecules, this presents no problem because the molecules are small enough to be placed in the field. For lanthanide upconverters, this is more difficult because the ions are typically contained in materials with grain sizes in the micrometer range. However, several groups have managed to make nanosized NaYF4 particles [67,68]. This offers the possibility of plasmonic enhancement for lanthanide upconverters and decreases the light intensity required for efficient upconversion. Alternatively, upconversion using sensitized triplet-triplet annihilation in organic molecules at moderate monochromatic excitation intensities increases the a-Si:H cell efficiency as well [46,56].

## Conclusions

In this paper, we have briefly reviewed upconversion for solar cells and have presented some relevant experimental results, focusing on the application of lanthanides in combination with wide-bandgap solar cells (a-Si:H). The proof-of-principle experiments that have been performed so far have shown that high intensities are needed to demonstrate upconversion for solar cells. Within the lanthanides, large steps in decreasing the necessary intensity are not expected. In the organic field, there is a rapid decrease in intensity needed for efficient upconversion, while conversion wavelengths are not appropriate yet.

External sensitization using quantum dots or options to enhance upconversion efficiencies by manipulating emission and excitation processes through plasmonic coupling may offer routes for successful upconversion deployment in solar cells. With further developments in these organic molecules, it remains to be seen if lanthanide upconverters, with plasmonic enhancement, or molecules in which TTA can be employed, will be the upconverter material for the future in wide-bandgap solar cells.

## Abbreviations

AM: air mass; APE: average photon energy; DSSC: dye-sensitized solar cell; ECE: external collection efficiency; FC-C: front converter and cell; IR: infrared; NIR: near infrared; PMMA: polymethylmethacrylate; PV: photovoltaics; QD: quantum dot; TTA: triplet-triplet annihilation.

## Competing interests

The authors declare that they have no competing interests.

## Authors’ contributions

RS, WvS, JR, and AM initiated and conceived this study. JdW, as a Ph.D. student in the groups of RS and AM under the cosupervision of JR and WvS, performed the experiments. WvS and JdW wrote the article. All authors read and approved the manuscript.
